# Knowledge About HPV and the HPV Vaccine: Observational Study on a Convenience Sample of Adolescents from Select Schools in Three Regions in Italy

**DOI:** 10.3390/vaccines13030227

**Published:** 2025-02-24

**Authors:** Laura Brunelli, Francesca Valent, Manola Comar, Barbara Suligoi, Maria Cristina Salfa, Daniele Gianfrilli, Franz Sesti, Giuseppina Capra, Alessandra Casuccio, Erik De Luca, Emily Bertola, Silvia Gazzetta, Lorenza Driul, Andrea Isidori, Patrizia Ferro, Nicolò Piazza, Palmira Immordino, Teresa Fasciana, Vincenzo Restivo

**Affiliations:** 1 Dipartimento di Medicina, Università degli Studi di Udine, 33100 Udine, Italy; gazzetta.silvia@spes.uniud.it; 2 SOC Accreditamento, Qualità e Rischio Clinico, Azienda Sanitaria Universitaria Friuli Centrale, 33100 Udine, Italy; erik.deluca@asufc.sanita.fvg.it; 3 SOC Igiene e Sanità Pubblica, Dipartimento di Prevenzione, Azienda Sanitaria Universitaria Friuli Centrale, 33100 Udine, Italy; francesca.valent@asufc.sanita.fvg.it (F.V.); emily.bertola@asufc.sanita.fvg.it (E.B.);; 4 Diagnostica Avanzata Microbiologica Traslazionale, IRCSS Burlo Garofolo, 34137 Trieste, Italy; manola.comar@burlo.trieste.it; 5 Dipartimento Universitario Clinico di Scienze Mediche Chirurgiche e Della Salute, Università degli Studi di Trieste, 34127 Trieste, Italy; 6 Centro Operativo AIDS, Dipartimento Malattie Infettive, Istituto Superiore di Sanità, 00161 Rome, Italy; barbara.suligoi@iss.it (B.S.); mariacristina.salfa@iss.it (M.C.S.); 7 Dipartimento di Medicina Sperimentale, Università Sapienza di Roma, 00185 Rome, Italy; daniele.gianfrilli@uniroma1.it (D.G.); franz.sesti@uniroma1.it (F.S.); andrea.isidori@uniroma1.it (A.I.); 8 Sezione di Microbiologia, Dipartimento PROMISE, Università di Palermo, 90127 Palermo, Italy; giuseppina.capra@unipa.it (G.C.); teresa.fasciana@unipa.it (T.F.); 9 Sezione di Igiene, Dipartimento PROMISE, Università di Palermo, 90127 Palermo, Italy; alessandra.casuccio@unipa.it (A.C.); patrizia.ferro@unipa.it (P.F.); nicolo.piazza@unipa.it (N.P.); palmira.immordino@unipa.it (P.I.); 10 SOC Ostetricia e Ginecologia, Azienda Sanitaria Universitaria Friuli Centrale, 33100 Udine, Italy; lorenza.driul@asufc.sanita.fvg.it; 11 Dipartimento di Medicina, Università Kore di Enna, 94100 Enna, Italy; vincenzo.restivo@unikore.it

**Keywords:** HPV, adolescents, prevention, public health, school

## Abstract

Background/Objectives: HPV is the most common sexually transmitted infectious agent worldwide and adolescents are at high risk of contracting HPV. The aim of our study was to find out how much adolescents know about the virus and its effects, and to obtain information on attitudes and behaviors regarding HPV vaccination to close these gaps. Methods: As part of the ESPRIT project, 598 lower secondary (11–14 years) and upper secondary (14–19 years) school students from three Italian regions were surveyed between December 2023 and March 2024 using a seven-question online questionnaire on awareness, knowledge, and attitudes about HPV and the HPV vaccine. Count and zero-inflation models were used to determine correlations between sexes, urban/suburban, province of residence, and school type with knowledge. Results: Lower secondary students believed that HPV causes HIV/AIDS (8.9%) or hepatitis C (3.0%) and rarely mentioned anal (21%) and oral sex (9.6%) as ways of transmission. Among upper secondary students, misconceptions were similar, with worrying rates of students stating that HPV only causes cancer in females (18%) or males (2.4%), and low rates of identifying transmission risk through anal (41%) and oral (34%) sex and genital contact (38%). The HPV vaccination rate was quite low (47% in lower secondary students, 61% in upper secondary students). In the regressions, sex, urban/suburban area, and province were the variables associated with higher levels of knowledge for lower secondary students; for upper secondary students, level of knowledge was associated with sex, urban/suburban area, school type, and province of residence. Conclusions: Awareness and knowledge of HPV and the HPV vaccine are low among Italian students in this study and reported vaccination coverage is below the national target. Coordinated efforts at the national level are needed to address this public health issue.

## 1. Introduction

The human papillomavirus (HPV) is recognized to be the cause of almost all cases of cervical cancer and a large proportion of other cancers of the oropharynx, vagina, vulva, penis, and anus in men and women [1], with a total of around 730,000 cases of cancer worldwide attributed to HPV in 2020 [2]. HPV is the most common sexually transmitted infectious agent worldwide with an adjusted global prevalence of 11.7% [3]. Although most infections are asymptomatic and resolve within a few years, HPV infection can lead to clinical conditions such as anogenital warts and the cancers mentioned above [1]. For this reason, the World Health Organization (WHO) called for a global strategy in 2020 to accelerate the elimination of cervical cancer as a public health problem by improving primary and secondary prevention and treatment [4].

The main risk factors for acquiring HPV infection are behaviors associated with sexual activity [1]. Youth and adolescents who engage in early sexual activity are most at risk due to their psychological predisposition to high-risk behaviors [5], lack of awareness and knowledge of the potential consequences [6,7,8,9], and inadequate access to prevention and health services [10,11,12]. In fact, according to the Health Belief Model (HBM), there is a clear association between improving knowledge and implementation of safe sexual and reproductive behaviors and uptake of HPV-specific vaccination [13,14], which is highly effective in preventing HPV-related diseases and cancers [15].

The current situation in Italy is not ideal for either vaccination or screening programs [16], perhaps also due to the recent pandemic we have experienced [17]. Since improving knowledge and promoting access to prevention and health services would improve outcomes related to HPV it is, therefore, important to know to what extent adolescents are aware of the virus and its effects, and to obtain information on young people’s sexual and reproductive health attitudes and behaviors regarding HPV vaccination in order to fill these gaps.

The aim of our study was to assess the awareness, knowledge, and vaccination practices of Italian adolescents regarding HPV.

## 2. Materials and Methods

### 2.1. The Research Instrument

**Figure 1:**
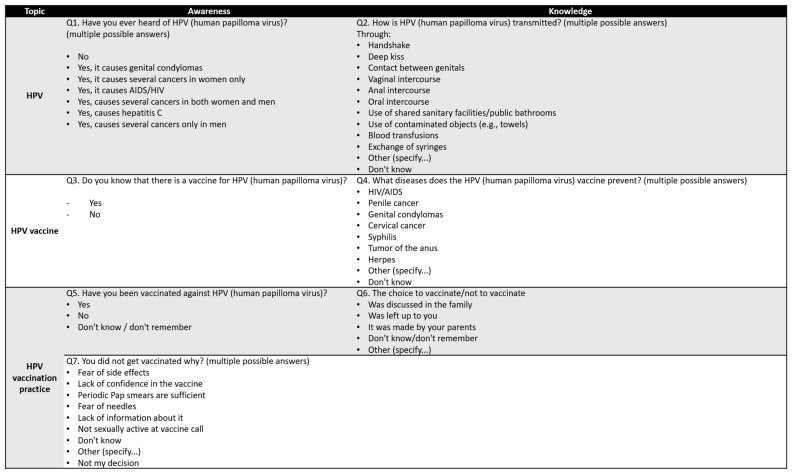
Questions on awareness, knowledge, and practice about HPV and HPV vaccination.

The research group conducting the project “Education in lower and upper secondary school and support of the network of adolescents reference persons for the prevention of HPV and other sexually transmitted infections (ESPRIT)” [18] funded by the CCM program (Centro nazionale per la prevenzione e il Controllo delle Malattie; Italian National Center for Disease Control and Prevention) of the Italian Ministry of Health, selected seven questions to be included in an online survey to investigate the awareness, knowledge, and practices of Italian adolescents specifically in relation to HPV. The questions (see Figure 1) were part of a broader survey that collected information about the participants, as previously described in the publication of the study protocol [18].

The seven questions related to awareness of the existence of HPV (Q1), knowledge of routes of transmission (Q2), awareness about the existence of a vaccine against HPV (Q3), knowledge of diseases prevented by the HPV vaccine (Q4), HPV vaccination practices (Q5), information on vaccination decision-making (Q6), and reasons for non-vaccination (Q7).

### 2.2. Data Collection

**Figure 2:**
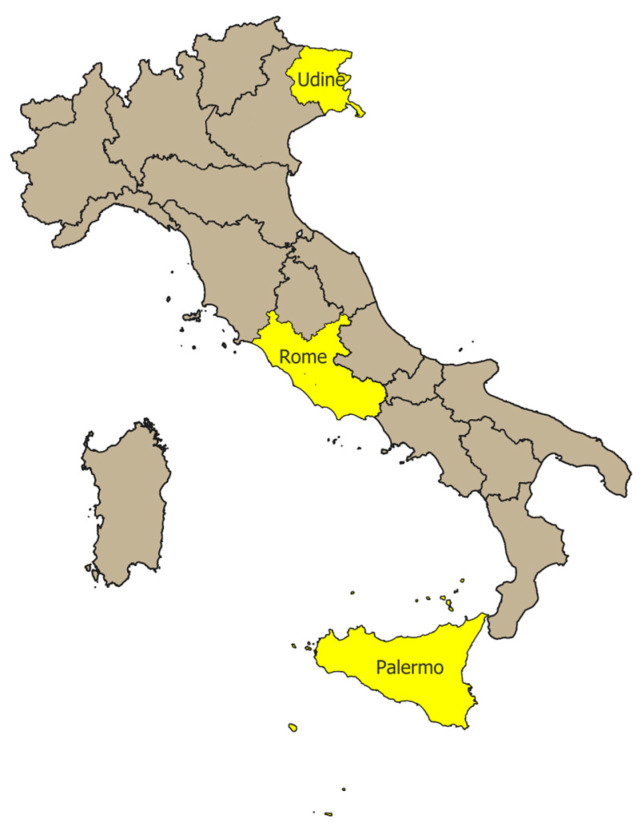
Italian regions and provinces participating in the ESPRIT project.

Adolescents attending one of the 10 lower secondary schools (age range 11–14 years) or 16 upper secondary schools (age range 14–19 years) in the provinces of Udine (northern Italy), Rome (central Italy), and Palermo (southern Italy) participating in the ESPRIT project were invited to participate in the survey (Figure 2). The selected provinces represented the three Italian macro-regions (North, Center, South) and corresponded to the locations of the participating partners that have been funded after the national call by the Italian Ministry of Health (CCM program). The schools were selected by the participating centers in collaboration with the local health authorities to include both urban and suburban areas, with the main city in each province considered urban and other villages considered suburban. To be eligible, schools had to agree to participate in the project and could not be involved in other STI/HPV or SRH information and education projects. The selected schools were first contacted by the regional contact person by letter and/or e-mail to introduce the project and to ask about the willingness of the institutions to participate. To this end, the head teachers were provided with background information and the objectives to be pursued, and the modalities and schedule of the subsequent intervention as part of the ESPRIT project were presented.

Participation was voluntary and free of charge. Adolescents completed the online survey themselves in their classroom after obtaining written parental consent and assent from minors. In some cases where the online platform was temporarily unavailable, the data were collected on paper and then entered into the database by the researchers on site. A member of the research team was always present during the collection of the questionnaires to ensure the validity of the data and minimize bias. Data collection took place after each participant was assigned a unique code to pseudo-anonymize the collected data. This paper presents the results of the baseline survey, which took place between December 2023 and March 2024, i.e., prior to the start of the educational activities offered as part of the ESPRIT project. The study was approved by the Regional Unique Ethic Committee of Friuli Venezia Giulia (CEUR-2023-Sper-34). The project was carried out with the technical and financial support of the Italian Ministry of Health—CCM.

### 2.3. Data Analysis

The data collected from lower and upper secondary school participants were analyzed separately, considering the different ages of adolescents and a possible simultaneous sexual debut (15–18 years is the average age of the first sexual encounter of Italian adolescents, as shown in a recent study by Fallucca et al. [19]), but also the fact that all older adolescents should already be offered the full HPV vaccination cycle free of charge at a national level (first offer at 11 years; second and further calls during following years) according to the Italian National Vaccination Plan [20]. HPV vaccination in Italy is offered by the local health authorities, who make an appointment by post or telephone. In certain cases, where there are specific contextual factors (i.e., hard-to-reach villages, availability of healthcare professionals and collaboration with schools), it is administered at school. A certain delay in the administration of the HPV vaccine in some regions cannot be ruled out.

Sociodemographic data such as school characteristics (e.g., urban/suburban, lower/upper secondary school, northern/central/southern Italy, type of upper secondary school) and participant characteristics (e.g., gender) were included as predictors in the model, while the total score was set as the dependent variable. The types of upper secondary schools considered reflect the three main types of schools that exist in Italy: academic schools (in which the education is mainly theoretical, with a specialization in a specific field, e.g., humanities and antiquity, mathematics and natural sciences, foreign languages, psychology and pedagogy, social sciences, fine arts), technical schools (in which the education provides both a broad theoretical training and a specialization in a specific field (e.g., economics, administration, technology, tourism, agronomy), often in combination with a three-/six-month internship in a company, association, or college), and vocational schools (this type of school provides secondary education with a focus on practical subjects (e.g., engineering, agriculture, gastronomy, technical assistance, crafts) and allows students to start looking for a job immediately after completing their education). None of these types of schools are likely to be single-sex schools in the national public system.

There were several possible answers to each question. If an answer was marked correctly, the student received +1, otherwise 0. If the answer was “I don’t know”, the score for the entire question was set to 0, as the student did not know the correct answer, even if they had not marked the wrong answer. The answers given by the students as strings (i.e., “Other”) were not considered when calculating the points. The total score was calculated as the sum of the scores for each valuable question, including Q1, Q2, and Q4. For every question, the frequency and probability of responses for every socio-demographic characteristic were calculated. In addition, the *p*-value and significance of the proportion test were calculated for each pair of sociodemographic characteristics. The minimum *p*-value was set at 0.05 and the test power had to be at least 0.8. For the analysis of the overall scores, each observation was weighted to obtain an inverse propensity weighting (IPW) for each linear combination of sociodemographic characteristics. However, for some linear combinations, there were no observations, because there were no schools for these combinations, which made an IPW impossible. Therefore, the data for these groups were generated using a generalized linear model (family quasipoisson) to minimize bias, and then the IPW was calculated. For each socio-demographic characteristic, the weighted Kruskal–Wallis test was calculated to demonstrate stochastic superiority. A t-test or ANOVA test was not used as the assumptions were not met. Finally, a weighted Poisson model with zero-inflation was calculated. First, the probability of receiving 0 points in the survey was assessed using a binomial model and then a Poisson model was calculated.

## 3. Results

### 3.1. Description of the Sample of Participants

The survey was completed by a total of 598 adolescents (44% response rate), of whom 135 (23%) attended lower secondary school and 463 (77%) attended upper secondary school. Students at upper secondary schools who took part were in the survey more frequently (49%) than students at lower secondary school (26%). Most of the participants were girls (344; 57%); the adolescents came from the provinces of Udine (277; 46%), Palermo (205; 34%), and Rome (116; 20%). The mean age of adolescents attending lower secondary education was 12 years; the proportion of girls was 55% (74/135), they studied in urban (71; 53%) and suburban contexts (64; 47%) in the only provinces of Palermo (84; 62%) and Udine (51; 38%) without a migrant background (133; 99% born in Italy). Among the adolescents attending upper secondary school, the mean age was 16 years; the prevalence of girls was 58% (270/463) and came from urban (279; 60%) and suburban contexts (184; 40%) in the provinces of Udine (226; 49%), Palermo (121; 26%), and Rome (116; 25%) with a slight prevalence of migrant background (28; 6% of them were not born in Italy).

### 3.2. Lower Secondary School Adolescents

Overall, half of the participants indicated that the existence of HPV was known among lower secondary school adolescents, although some adolescents believed that HPV causes HIV/AIDS (12; 8.9%), cancer only in females (10; 7.4%), cancer only in males (2; 1.5%), or hepatitis C (4; 3.0%). In general, adolescents who reported never having heard of HPV were evenly distributed between boys and girls and between urban and suburban contexts but were slightly more common, although not significantly so, in the province of Palermo (*p*-value = 0.673). Knowledge of HPV transmission methods was very low among these younger adolescents. The best recognized transmission method was contact between the genitals (57; 42%) and vaginal intercourse (56; 41%), although less than half of the participants correctly identified this. Other common transmission methods such as anal intercourse (29; 21%) and oral intercourse (13; 9.6%) were hardly recognized. There were differences between participants who admitted not knowing the transmission methods, especially among those who lived in urban areas (*p*-value = 0.049).

Nonetheless, awareness of the existence of the HPV vaccine was quite high among younger adolescents (109; 81%), even if it appeared to be higher among girls (64; 86%), adolescents living in urban areas (61; 86%) and in the province of Udine (44; 86%), although this was not statistically significant. Knowledge of the diseases that the HPV vaccine can prevent was very low, with the main correct answers given in only a few cases: genital condylomas (17; 13%), cervical cancer (17; 13%), penile cancer (28; 21%), and tumors of the anus (22; 16%). On the other hand, the adolescents expected that the HPV vaccine would protect against HIV/AIDS (38; 21%) and herpes (18; 14%) to about the same extent. Table 1 shows the responses of lower secondary school adolescents to these first four questions, which tested awareness and knowledge of HPV and the HPV vaccine.

When adolescents in lower secondary school were asked about their HPV vaccination, 47% of them (n = 64) stated that they had received the HPV vaccine, while 30% (n = 40) had not and 21% (n = 29) could not remember or did not know the answer. HPV vaccination was slightly more frequently, but not significantly, reported by girls (37; 50%) and by adolescents living in the suburbs (32; 50%) of the province of Udine (25; 49%). In most cases, adolescents reported that the decision about HPV vaccination was made by their parents (58; 43%), and few reported that they had discussed the issue with their families (19; 14%). Participants from urban contexts were more likely to report that they could not recall the decision-making process for their vaccination (*p*-value = 0.02). The main reasons for not being vaccinated against HPV were mostly unknown to the adolescents (23; 17%).

### 3.3. Upper Secondary School Adolescents

In general, two-thirds of upper secondary school participants reported some awareness about HPV, although some of them believed that HPV causes cancer only in females (83; 18%) or only in males (11; 2.4%), and that it causes HIV/AIDS (89; 19%) or hepatitis C (21; 4.5%). Unawareness was greater among boys (40%; *p*-value = 0.018) who attended a technical secondary school (42%; *p*-value = 0.013). Girls were more likely than others to believe that HPV only causes cancer in females (24%; *p*-value < 0.001), as were students from Palermo (25%; *p*-value = 0.024), while participants living in urban areas were more likely to know that HPV causes cancer in both males and females (32%; *p*-value = 0.032). Participants from Rome were more likely to be aware that there is a causal relationship between HPV and genital condylomas (17%; *p*-value = 0.032). Knowledge of HPV transmission differed between the transmission methods: vaginal intercourse (299; 65%), anal intercourse (190; 41%), genital contact (178; 38%), and oral intercourse (158; 34%). Several differences were found between sexes, living areas, provinces, and school types for vaginal intercourse, genital contact, syringe sharing, blood transfusion, and the use of shared sanitary facilities/public toilets as possible transmission methods (see Table 2).

Adolescents’ awareness of the existence of a vaccine against HPV was quite high (406; 88%) and significantly higher among girls (92%; *p*-value = 0.003) and academic upper secondary school students (92%; *p*-value = 0.016). Differences in awareness of this prevention measure were also found in relation to the province of residence, where Palermo performed better, but without reaching statistical significance. No differences were found in the urban/suburban areas. Knowledge of the diseases prevented by the HPV vaccine was quite poor: only 171 adolescents (37%) were able to correctly identify cervical cancer, and even fewer of them knew that it was genital condylomas (77; 17%), penile cancer (109; 24%), and anal cancer (56; 12%). On the other hand, many adolescents (203; 44%) believed that the HPV vaccine can prevent HIV/AIDS. Numerous differences were found between sexes, e.g., in knowledge of the consequences of penile (*p*-value = 0.009) and cervical cancer (*p*-value < 0.001), with boys scoring higher in knowledge of genital condyloma and girls scoring higher in knowledge of cervical cancer. Academic students were more likely than others to know that HPV causes genital condyloma (22%; *p*-value = 0.025) and cervical cancer (43%; *p*-value = 0.038). Participants living in urban areas were more likely to recognize genital condyloma as an HPV-related disease (21%; *p*-value = 0.005), while adolescents from suburban areas were more likely to believe that HIV/AIDS is caused by HPV (50%; *p*-value = 0.038). Table 2 shows the responses of upper secondary school adolescents to the first four questions testing awareness and knowledge of HPV and the HPV vaccine.

Upper secondary school students recalled in 61% of cases (n = 281) that they had been administered the HPV vaccine. This event was particularly well known among girls (68%, *p*-value = 0.001), adolescents from the province of Udine (66%, *p*-value < 0.001), coming from an urban area (68%, *p*-value < 0.001), and attending an academic school (70%, *p*-value < 0.001). In most cases, the decision about HPV vaccination was made by parents (246; 53%), especially in the province of Udine (61%; *p*-value < 0.001) and in urban areas (170; 61%, *p*-value < 0.001). The reasons for missing the HPV vaccination were largely unknown to the adolescents themselves.

### 3.4. Determinants for a Higher Level of Knowledge About HPV and the HPV Vaccine

Students in upper secondary school generally achieved a score of 11.2 ± 5.6, while students in lower secondary school achieved a mean score of 8.0 ± 6.1. The scores of the former differed greatly by sex area, and those of the latter by sex, area, province of residence, and type of school. The count model analysis showed that girls and students from urban areas, particularly in the province of Palermo, achieved the highest knowledge scores for lower secondary school participants. The same model analysis showed that sex, area, school type, and province of residence influenced knowledge levels of HPV and the HPV vaccine among upper secondary school students. Girls, students from urban areas, particularly from the province of Rome, and students attending academic schools performed better. The zero-inflated model obtained similar results, which are reported in Table 3 along with the regression analysis of the count model.

## 4. Discussion

This study investigated the awareness, knowledge, and vaccination practices of a sample of Italian adolescents attending lower and upper secondary school regarding HPV and the HPV vaccine. In general, we found that awareness and knowledge of HPV is low, although it improves somewhat as adolescents become older. Some of the most common methods of HPV transmission were not recognized by the adolescents, e.g., oral and anal sex. Regarding HPV vaccination, the level of knowledge of adolescents in both school levels was quite good, although some misconceptions about the diseases against which this vaccine protects are still widespread. The HPV vaccination rates reported by adolescents were below the desirable level, and this seems to increase with the age of the respondents, but they still indicated in most cases that they were not involved in the parental decision-making process.

The general level of awareness about HPV that we found among adolescents in lower secondary school (50%) and upper secondary school (66%) is similar to the 68% found by colleagues in southern Italy [11], but differs markedly from the 71% found in a study of lower secondary school students in Palermo [21] and the 87% found in the same city [19], although the latter study surveyed larger class groups (university and upper secondary school students). The low general knowledge level found in this study confirms what has already been reported in the literature by Italian [6] and French colleagues [9], as well as the higher knowledge among girls [7,9], even when it comes to sexually transmitted infections in general [10]. Our results also confirmed some differences found in Greece related to urban/rural areas [22], and in Italy related to school type [10]. No information could be compared on other determinants reported in the literature, such as income and nationality [10,21], migration background [7,10], and age of first contact with sexual and reproductive health education [10]. Sexual intercourse was generally recognized as a potential method of transmission for HPV infection, as previously reported for lower secondary school students [21] and upper secondary school students [6], although knowledge of vaginal, oral, and anal intercourses was not examined separately in these papers. The warning signs that emerge from the analysis of adolescents’ knowledge should not discount the fact that many adolescents still believe that the HPV vaccine can protect against HIV/AIDS, as reported by Brunelli et al. [7]. With regard to this particular topic, it cannot be ruled out that there may be misunderstandings and confusion between the terms HPV and HIV among students. Nonetheless, consideration of the overall low levels of complacency resulting from analyzing data from this group of individuals who are close to or at the age of sexual debut [19] is more than a cause for concern.

Adolescents’ awareness of their HPV vaccination status and the resulting vaccination coverage was quite low, confirming national data [11,16]. Although this finding in our study could also be partly due to adolescents being unaware of their vaccination status because they were not involved in the decision-making process, the result remains concerning. Nonetheless, delays in the implementation of the vaccination call by local health authorities, which is expected for boys and girls aged 11 in Italy, cannot be ruled out and this may have partly contributed to the low vaccination rates, especially among lower secondary school students who may not have been called yet. Much remains to be achieved to reduce complacency in general, to include the male perspective in education about HPV and HPV vaccination [7], to involve fathers in the conversation [23] and, for example, to inform boys if a member of their family or social network has been vaccinated against HPV, or to raise concerns about the sexual and reproductive health of their female partners [24]. Nonetheless, the characteristics of target groups that are less likely to be vaccinated need to be considered, not only in terms of known health determinants such as gender, income, and place of residence [21], but also considering other factors such as health literacy [19]. Since vaccine hesitancy is also related to individual cognitive characteristics [25], a fusion of competencies and areas of activity between social and cognitive sciences is desirable. Finally, greater involvement of adolescents in the familiar discussion about HPV vaccination is needed, as also called for by Lefevre et al. [26], as the low participation reported in this study was not an isolated case [7], and better results could be achieved at a national level [6]. This applies to older adolescents, but younger adolescents should also be included in the conversation, as the Italian National Vaccination Plan offers HPV vaccination for the first time at age 11 [20]; therefore, adolescents themselves need to be better informed and involved in the decision-making process, as this has been shown to be effective [27].

To improve vaccination rates, the role of families in HPV prevention should be strengthened and the low knowledge of parents about HPV reported by colleagues [6] should be addressed, as it was found that parents’ willingness to have adolescents vaccinated against HPV was related to their higher knowledge of HPV vaccination and their perceived greater susceptibility to HPV [13]. Some interventions for this target group have already been successfully implemented [28,29] and others will be reported on shortly. This includes the planned intervention as part of the CCM project that our group is working on [18].

However, the key role of schools in ensuring equitable sexual and reproductive health education is undeniable, and the effectiveness of school-based interventions on this issue has been reported [21,22], including the direct delivery of vaccination in schools where coverage is particularly low [21]. To achieve better teacher engagement and ensure the effectiveness and sustainability of interventions, key reported barriers such as lack of information about HPV, negative attitudes towards the vaccine and fear of parental refusal of HPV vaccination need to be addressed. Teachers should be supported by emphasizing the perceived benefits, awareness of HPV, the school and the perception of the important role of teachers in promoting HPV vaccination [30].

There may be greater exposure to public health initiatives and services targeting young people in an urban setting, which could explain the differences in knowledge and immunization rates. Nonetheless, these variables, along with school type and province of residence, should be carefully considered when planning and implementing interventions to improve knowledge about HPV and the HPV vaccine, as well as when conducting vaccination campaigns to ensure equal treatment of all adolescents. Further efforts need to be made in relation to hard-to-reach populations, i.e., adolescents and families living in suburban areas and students attending technical and vocational schools, where knowledge gaps and therefore risks are greater.

Nonetheless, some other stakeholders could be more involved in the conversation about this public health issue, such as pediatric dentists, who are already positive about their role in preventing oropharyngeal cancer and have a good attitude when it comes to discussing sensitive topics with their patients [31]. Given the general population’s poor knowledge of the link between HPV and oropharyngeal cancer [32], these health professionals could help reduce parental complacency in an environment that could be perceived as more neutral and health oriented. In any case, the confidence of all healthcare professionals to discuss this topic with parents and families should be further encouraged and trained during the study years [33]. Nevertheless, institutions and families play a crucial and very important role in educating young people and overcoming social and cultural barriers [11]. Indeed, the key role of parents and school in such a scenario is widely recognized, and their involvement and commitment to improving adolescents’ sexual and reproductive health is called for by many authors [6,10,19,21,30,34] and institutions [35,36,37].

### Limitations

Our study has several limitations that should be taken into account when considering our results. First, some subcategories of respondents (i.e., lower secondary school students from Rome and academic suburban upper secondary school students from Udine) were not adequately represented in our sample, so the generalizability of the results may have suffered from this lack of information. Moreover, the selection of the three Italian regions involved in the project and the selection of the schools that participated in the project was opportunistic, so that this selection could not be representative of the Italian adolescent population. Second, for HPV vaccination, we could only rely on self-reported data as the retrieval of the HPV vaccination list from the records of local health authorities is subject to data protection. Therefore, vaccination rates may have been either overestimated due to social desirability bias or underestimated due to recall bias. Third, no family- or teacher-level variables were collected, so the differences found would benefit from further studies that also take these two elements into account. For example, adolescents whose siblings have already had received the HPV vaccine may have higher levels of knowledge, and schools where teachers were offered information/education programs on vaccination may have talked to their students about the topic. However, differences in the school system between provinces or schools are not expected, as all schools studied are public and, therefore, have to follow the same national guidelines. Therefore, the differences found may be partly due to other contextual factors that should be investigated further. Finally, we were not able to collect questionnaires from students whose parents refused to participate in the study, and we cannot say how their contribution would have changed our observations.

## 5. Conclusions

In conclusion, awareness and knowledge of HPV and the HPV vaccine among the studied group of Italian lower and upper secondary school students is low and the reported vaccination rate is below the national target. Efforts should be made in relation to this public health problem, considering adolescents, their parents, and the school as essential target groups for a coordinated intervention that needs to be standardized at national level.

## Figures and Tables

**Table 1:** Responses of lower secondary school adolescents to questions 1–4 on awareness and knowledge about HPV and the HPV vaccine, by sex, urban/suburban, province of residence, and overall.

Answers	Overall (N = 135)	Sex (n (%))			Urban/Suburban (n (%))		Province of Residence (n (%))	
		Boys (N = 59)	Girls (N = 74)	Other (N = 2)	Urban (N = 71)	Suburban (N = 64)	Udine (N = 51)	Palermo (N = 84)
Awareness about HPV—Q1: Have you ever heard of HPV (human papilloma virus)?								
No	68 (50%)	30 (51%)	37 (50%)	1(50%)	36 (51%)	32 (50%)	24 (47%)	44 (52%)
Yes, it causes several cancers in both women and men	33 (24%)	11 (19%)	22 (30%)	0 (0%)	20 (28%)	13 (20%)	**18 (35%) ^§^**	**15 (18%) ^§^**
Yes, it causes genital condylomas	18 (13%)	11 (19%)	7 (9.5%)	0 (0%)	12 (17%)	6 (9.4%)	3 (5.9%)	15 (18%)
Yes, it causes AIDS/HIV	12 (8.9%)	6 (10%)	6 (8.1%)	0 (0%)	6 (8.5%)	6 (9.4%)	5 (9.8%)	7 (8.3%)
Yes, it causes several cancers in women only	10 (7.4%)	6 (10%)	4 (5.4%)	0 (0%)	4 (5.6%)	6 (9.4%)	2 (3.9%)	8 (9.5%)
Yes, it causes hepatitis C	4 (3.0%)	2 (3.4%)	2 (2.7%)	0 (0%)	2 (2.8%)	2 (3.1%)	2 (3.9%)	2 (2.4%)
Yes, it causes several cancers only in men	2 (1.5%)	1 (1.7%)	1 (1.4%)	0 (0%)	1 (1.4%)	1 (1.6%)	1 (2.0%)	1 (1.2%)
missing	5 (3.7%)	1 (1.7%)	3 (4.1%)	1(50%)	1 (1.4%)	4 (6.3%)	2 (3.9%)	3 (3.6%)
Knowledge about HPV—Q2: How is HPV (human papilloma virus) transmitted?								
Contact between genitals *	57 (42%)	23 (39%)	34 (46%)	0 (0%)	31 (44%)	26 (41%)	18 (35%)	39 (46%)
Vaginal intercourse *	56 (41%)	22 (37%)	34 (46%)	0 (0%)	31 (44%)	25 (39%)	21 (41%)	35 (42%)
Anal intercourse *	29 (21%)	15 (25%)	14 (19%)	0 (0%)	19 (27%)	10 (16%)	13 (25%)	16 (19%)
Deep kiss *	17 (13%)	4 (6.8%)	13 (18%)	0 (0%)	11 (15%)	6 (9.4%)	5 (9.8%)	12 (14%)
Use of shared sanitary facilities/public bathrooms	14 (10%)	5 (8.5%)	9 (12%)	0 (0%)	7 (9.9%)	7 (11%)	2 (3.9%)	12 (14%)
Use of contaminated objects (e.g., towels) *	13 (9.6%)	5 (8.5%)	8 (11%)	0 (0%)	6 (8.5%)	7 (11%)	3 (5.9%)	10 (12%)
Oral intercourse *	13 (9.6%)	5 (8.5%)	8 (11%)	0 (0%)	8 (11%)	5 (7.8%)	3 (5.9%)	10 (12%)
Exchange of syringes *	13 (9.6%)	4 (6.8%)	9 (12%)	0 (0%)	8 (11%)	5 (7.8%)	5 (9.8%)	8 (9.5%)
Blood transfusions *	13 (9.6%)	5 (8.5%)	8 (11%)	0 (0%)	9 (13%)	4 (6.3%)	5 (9.8%)	8 (9.5%)
Handshake	3 (2.2%)	1 (1.7%)	2 (2.7%)	0 (0%)	3 (4.2%)	0 (0%)	1 (2.0%)	2 (2.4%)
Don’t know	44 (33%)	23 (39%)	20 (27%)	1(50%)	**29 (41%) ^§^**	**15 (23%) ^§^**	22 (43%)	22 (26%)
missing	7 (5.2%)	3 (5.1%)	3 (4.1%)	1(50%)	0 (0%)	7 (11%)	4 (7.8%)	3 (3.6%)
Awareness about HPV vaccine—Q3: Do you know that there is a vaccine for HPV (Human Papilloma Virus)?								
Yes	109 (81%)	45 (76%)	64 (86%)	0 (0%)	61 (86%)	48 (75%)	44 (86%)	65 (77%)
No	22 (16%)	12 (20%)	9 (12%)	1(50%)	9 (13%)	13 (20%)	6 (12%)	16 (19%)
missing	4 (3.0%)	2 (3.4%)	1 (1.4%)	1(50%)	1 (1.4%)	3 (4.7%)	1 (2.0%)	3 (3.6%)
Knowledge about HPV vaccine—Q4: What diseases does the HPV (Human Papilloma Virus) vaccine prevent?								
Genital condylomas *	17 (13%)	8 (14%)	9 (12%)	0 (0%)	11 (15%)	6 (9.4%)	4 (7.8%)	13 (15%)
HIV/AIDS	28 (21%)	14 (24%)	14 (19%)	0 (0%)	11 (15%)	17 (27%)	6 (12%)	22 (26%)
Penile cancer *	28 (21%)	8 (14%)	20 (27%)	0 (0%)	19 (27%)	9 (14%)	8 (16%)	20 (24%)
Tumor of the anus *	22 (16%)	5 (8.5%)	16 (22%)	1 50%)	15 (21%)	7 (11%)	6 (12%)	16 (19%)
Herpes	19 (14%)	5 (8.5%)	14 (19%)	0 (0%)	11 (15%)	8 (13%)	2 (3.9%)	17 (20%)
Cervical cancer *	17 (13%)	3 (5.1%)	14 (19%)	0 (0%)	7 (9.9%)	10 (16%)	4 (7.8%)	13 (15%)
Syphilis	1 (0.7%)	1 (1.7%)	0 (0%)	0 (0%)	0 (0%)	1 (1.6%)	0 (0%)	1 (1.2%)
Don’t know	36 (27%)	14 (24%)	22 (30%)	0 (0%)	21 (30%)	15 (23%)	36 (71%)	0 (0%)
Other	28 (21%)	16 (27%)	12 (16%)	0 (0%)	**22 (31%) ^§^**	**6 (9.4%) ^§^**	0 (0%)	28 (33%)
missing	4 (3.0%)	2 (3.4%)	1 (1.4%)	1(50%)	0 (0%)	4 (6.3%)	1 (2.0%)	3 (3.6%)

* Correct option; in bold: statistically different values when <0.05 ^§^.

**Table 2:** Responses by upper secondary school adolescents to questions 1–4 on awareness and knowledge of HPV and the HPV vaccine, by sex, urban/suburban, province of residence and overall.

Answers	Overall (N = 463)	Sex (n (%))			Urban/Suburban (n (%))		Province of Residence (n (%))			Type of Upper Secondary School (n (%))		
		Boys (N = 185)	Girls (N = 270)	Other (N = 8)	Urban (N = 279)	Suburban (N = 184)	Udine (N = 226)	Rome (N = 116)	Palermo (N = 121)	Academic (N = 206)	Vocational (N = 114)	Technical (N = 143)
Awareness about HPV—Q1: Have you ever heard of HPV (human papilloma virus)?												
No	158 (34%)	**74 (40%) ^§^**	**78 (29%) ^§^**	**6 (75%) ^§^**	88 (32%)	70 (38%)	70 (31%)	45 (39%)	43 (36%)	**56 (27%) ^§^**	**42 (37%) ^§^**	**60 (42%) ^§^**
Yes, it causes several cancers in both women and men *	130 (28%)	44 (24%)	85 (31%)	1 (13%)	**89 (32%) ^§^**	**41 (22%) ^§^**	65 (29%)	33 (28%)	32 (26%)	69 (33%)	25 (22%)	36 (25%)
Yes, it causes AIDS/HIV	89 (19%)	39 (21%)	50 (19%)	0 (0%)	47 (17%)	42 (23%)	41 (18%)	23 (20%)	25 (21%)	37 (18%)	26 (23%)	26 (18%)
Yes, it causes several cancers in women only	83 (18%)	**19 (10%) ^^^**	**64 (24%) ^^^**	**0 (0%) ^^^**	49 (18%)	34 (18%)	**40 (18%) ^§^**	**13 (11%) ^§^**	**30 (25%) ^§^**	40 (19%)	20 (18%)	23 (16%)
Yes, it causes genital condylomas *	52 (11%)	17 (9.2%)	34 (13%)	1 (13%)	36 (13%)	16 (8.7%)	**24 (11%) ^§^**	**20 (17%) ^§^**	**8 (6.6%) ^§^**	19 (9.2%)	17 (15%)	16 (11%)
Yes, it causes hepatitis C	21 (4.5%)	12 (6.5%)	9 (3.3%)	0 (0%)	9 (3.2%)	12 (6.5%)	17 (7.5%)	2 (1.7%)	2 (1.7%)	6 (2.9%)	4 (3.5%)	11 (7.7%)
Yes, it causes several cancers only in men	11 (2.4%)	8 (4.3%)	3 (1.1%)	0 (0%)	10 (3.6%)	1 (0.5%)	7 (3.1%)	0 (0%)	4 (3.3%)	8 (3.9%)	1 (0.9%)	2 (1.4%)
Knowledge about HPV—Q2: How is HPV (human papilloma virus) transmitted?												
Vaginal intercourse *	299 (65%)	115 (62%)	180 (67%)	4 (50%)	179 (64%)	120 (65%)	145 (64%)	69 (59%)	85 (70%)	**145 (70%) ^§^**	**72 (63%) ^§^**	**82 (57%) ^§^**
Anal intercourse *	190 (41%)	75 (41%)	111 (41%)	4 (50%)	116 (42%)	74 (40%)	98 (43%)	42 (36%)	50 (41%)	95 (46%)	45 (39%)	50 (35%)
Contact between genitals *	178 (38%)	**52 (28%)^^^**	**124 (46%)^^^**	**2 (25%)^^^**	107 (38%)	71 (39%)	87 (38%)	45 (39%)	46 (38%)	81 (39%)	45 (39%)	52 (36%)
Oral intercourse *	158 (34%)	61 (33%)	94 (35%)	3 (38%)	97 (35%)	61 (33%)	81 (36%)	37 (32%)	40 (33%)	72 (35%)	40 (35%)	46 (32%)
Exchange of syringes *	114 (25%)	47 (25%)	66 (24%)	1 (13%)	**82 (29%) ^§^**	**32 (17%) ^§^**	**66 (29%) ^§^**	**19 (16%) ^§^**	**29 (24%) ^§^**	**64 (31%) ^§^**	**20 (18%) ^§^**	**30 (21%) ^§^**
Blood transfusions *	107 (23%)	47 (25%)	58 (21%)	2 (25%)	**77 (28%) ^§^**	**30 (16%) ^§^**	**66 (29%) ^§^**	**17 (15%) ^§^**	**24 (20%) ^§^**	**59 (29%) ^§^**	**21 (18%) ^§^**	**27 (19%) ^§^**
Use of contaminated objects (e.g., towels) *	48 (10%)	23 (12%)	24 (8.9%)	1 (13%)	34 (12%)	14 (7.6%)	26 (12%)	8 (6.9%)	14 (12%)	27 (13%)	7 (6.1%)	14 (9.8%)
Deep kiss *	46 (9.9%)	21 (11%)	23 (8.5%)	2 (25%)	29 (10%)	17 (9.2%)	26 (12%)	10 (8.6%)	10 (8.3%)	23 (11%)	12 (11%)	11 (7.7%)
Use of shared sanitary facilities /public bathrooms	26 (5.6%)	8 (4.3%)	15 (5.6%)	3 (38%)	**21 (7.5%) ^§^**	**5 (2.7%) ^§^**	16 (7.1%)	5 (4.3%)	5 (4.1%)	16 (7.8%)	4 (3.5%)	6 (4.2%)
Handshake	2 (0.4%)	0 (0%)	1 (0.4%)	1 (13%)	1 (0.4%)	1 (0.5%)	2 (0.9%)	0 (0%)	0 (0%)	0 (0%)	1 (0.9%)	1 (0.7%)
Other	6 (1.3%)	3 (1.6%)	2 (0.7%)	1 (13%)	3 (1.1%)	3 (1.6%)	2 (0.9%)	3 (2.6%)	1 (0.8%)	0 (0%)	2 (1.8%)	4 (2.8%)
Don’t know	108 (23%)	48 (26%)	58 (21%)	2 (25%)	71 (25%)	37 (20%)	52 (23%)	33 (28%)	23 (19%)	42 (20%)	27 (24%)	39 (27%)
*missing*	1 (0.2%)	0 (0%)	1 (0.4%)	0 (0%)	0 (0%)	1 (0.5%)	1 (0.4%)	0 (0%)	0 (0%)	0 (0%)	0 (0%)	1 (0.7%)
Awareness about HPV vaccine—Q3: Do you know that there is a vaccine for HPV (human papilloma virus)?												
Yes	406 (88%)	**152 (82%) ^^^**	**248 (92%) ^^^**	**6 (75%) ^^^**	246 (88%)	160 (87%)	195 (86%)	100 (86%)	111 (92%)	**189 (92%) ^^^**	**92 (81%) ^^^**	**125 (87%) ^^^**
No	57 (12%)	33 (18%)	22 (8.1%)	2 (25%)	33 (12%)	24 (13%)	31 (14%)	16 (14%)	10 (8.3%)	17 (8.3%)	22 (19%)	18 (13%)
Knowledge about HPV vaccine—Q4: What diseases does the HPV (human papilloma virus) vaccine prevent?												
HIV/AIDS	203 (44%)	77 (42%)	121 (45%)	5 (63%)	**111 (40%) ^§^**	**92 (50%) ^§^**	94 (42%)	53 (46%)	56 (46%)	80 (39%)	57 (50%)	66 (46%)
Cervical cancer *	171 (37%)	**41 (22%) ^^^**	**127 (47%) ^^^**	**3 (38%) ^^^**	102 (37%)	69 (38%)	81 (36%)	39 (34%)	51 (42%)	**88 (43%) ^§^**	**41 (36%) ^§^**	**42 (29%) ^§^**
Penile cancer *	109 (24%)	**56 (30%) ^§^**	**52 (19%) ^§^**	**1 (13%) ^§^**	65 (23%)	44 (24%)	46 (20%)	30 (26%)	33 (27%)	51 (25%)	29 (25%)	29 (20%)
Genital condylomas *	77 (17%)	31 (17%)	46 (17%)	0 (0%)	**58 (21%) ^§^**	**19 (10%) ^§^**	43 (19%)	18 (16%)	16 (13%)	**45 (22%) ^§^**	**15 (13%) ^§^**	**17 (12%) ^§^**
Tumor of the anus *	56 (12%)	28 (15%)	27 (10%)	1 (13%)	33 (12%)	23 (13%)	26 (12%)	17 (15%)	13 (11%)	19 (9.2%)	18 (16%)	19 (13%)
Syphilis	38 (8.2%)	17 (9.2%)	19 (7.0%)	2 (25%)	29 (10%)	9 (4.9%)	25 (11%)	8 (6.9%)	5 (4.1%)	22 (11%)	7 (6.1%)	9 (6.3%)
Herpes	34 (7.3%)	12 (6.5%)	20 (7.4%)	2 (25%)	21 (7.5%)	13 (7.1%)	20 (8.8%)	6 (5.2%)	8 (6.6%)	14 (6.8%)	11 (9.6%)	9 (6.3%)
Other	65 (14%)	34 (18%)	29 (11%)	2 (25%)	48 (17%)	17 (9.2%)	38 (17%)	20 (17%)	7 (5.8%)	28 (14%)	13 (11%)	24 (17%)
*missing*	1 (0.2%)	0 (0%)	1 (0.4%)	0 (0%)	1 (0.4%)	0 (0%)	1 (0.4%)	0 (0%)	0 (0%)	0 (0%)	0 (0%)	1 (0.7%)

* Correct option; in bold: statistically different values when <0.05 ^§^ or <0.001 ^^^.

**Table 3:** Profile of students with higher levels of knowledge about HPV and the HPV vaccine resulting from the regression model analysis.

Lower Secondary School Students									
Model		Count Model				Zero-Inflation Model			
		Estimate	Standard Error	z-Value	*p*-Value	Estimate	Standard Error	z-Value	*p*-Value
(Intercept)		2.25	0.05	42.48	**<0.001**	−1.15	0.24	−4.87	**<0.001**
Sex	Girls	-	-	-	-	-	-	-	-
	Boys	−0.11	0.05	−2.42	**0.015**	−0.11	0.23	−0.47	0.641
	Other	−0.23	0.30	−0.75	0.450	−12.80	1040.82	−0.01	0.990
Area	Suburban	-	-	-	-	-	-	-	-
	Urban	0.20	0.05	4.08	**<0.001**	0.81	0.24	3.43	**<0.001**
Province of residence	Udine	-	-	-	-	-	-	-	-
	Palermo	0.17	0.05	7.86	**<0.001**	−0.64	0.24	−2.69	**0.007**
**Upper Secondary School Students**									
**Model**		**Count Model**				**Zero-Inflation Model**			
		**Estimate**	**Standard Error**	**z-Value**	***p*-Value**	**Estimate**	**Standard Error**	**z-Value**	***p*-Value**
(Intercept)		2.62	0.01	204.95	**<0.001**	−5.30	0.38	−14.00	**<0.001**
Sex	Girls	-	-	-	-	-	-	-	-
	Boys	−0.12	0.01	−12.11	**<0.001**	0.34	0.18	1.91	0.056
	Other	−0.41	0.14	−3.01	**0.003**	1.15	1.15	1.01	0.315
Area	Suburban	-	-	-	-	-	-	-	-
	Urban	−0.14	0.01	−14.07	**<0.001**	0.13	0.18	0.72	0.468
Province of residence	Udine	-	-	-	-	-	-	-	-
	Rome	0.12	0.01	9.69	**<0.001**	0.90	0.20	4.45	**<0.001**
	Palermo	0.01	0.01	0.59	0.56	−0.83	0.30	−2.80	**0.005**
Type of school	Academic	-	-	-	-	-	-	-	-
	Technical	−0.16	0.01	−13.15	**<0.001**	2.42	0.34	7.16	**<0.001**
	Vocational	−0.13	0.01	−11.44	**<0.001**	1.22	0.37	3.32	**<0.001**

In bold: statistically different values.

## Data Availability

All data generated or analyzed during this study are included in this published article.
